# Syncope and the Inability to Move: Was It the Magnesium?

**DOI:** 10.7759/cureus.39868

**Published:** 2023-06-02

**Authors:** David R Perkins, Omar Kandah, Larissa Check, Robert Sherertz

**Affiliations:** 1 Internal Medicine, Grand Strand Medical Center, Myrtle Beach, USA

**Keywords:** proton-pump inhibitors (ppi), magnesium, movement disorders, syncope, electrolyte abnormalities

## Abstract

Proton pump inhibitors (PPIs) were clinically introduced more than 30 years ago and have been a very safe and effective agent for the treatment of a variety of different acid-base disorders. PPIs work by inhibiting the final step in gastric acid synthesis production by covalently binding to the (H+,K+)-ATPase enzyme system at the level of the gastric parietal cells leading to the irreversible inhibition of gastric acid secretion until new enzymes are produced. This inhibition is useful in a wide variety of disorders, which include, but are not limited to, gastroesophageal reflux disease (GERD), peptic ulcer disease, erosive esophagitis, *Helicobacter pylori *infection, and pathological hypersecretory disorders. Despite PPIs’ overall excellent safety profile, PPIs have raised concerns about both short- and long-term complications including multiple electrolyte derangements that can lead to life-threatening situations. We present a case of a 68-year-old male who presented to the emergency department after a syncopal episode with profound weakness and was found to have undetectable magnesium levels secondary to long-term omeprazole use. This case report highlights how important it is for clinicians to be aware of these electrolyte disturbances and the importance of monitoring electrolytes while being on these medications.

## Introduction

Magnesium is a very important electrolyte in regulating cellular processes. Normal levels of serum magnesium range from 1.46 to 2.68 mg/dL [1-3]. This divalent cation plays a vital role in cardiac function, skeletal muscle, nerve conduction, and many cellular transport mechanisms. Levels of magnesium less than 1.46 mg/dL can lead to prolonged corrected QT (QTc); tremors; seizures; stupor; generalized weakness or paralysis; or, in the worst case, death [1,2]. Pathological causes for hypomagnesemia include difficulty absorbing magnesium or wasting magnesium via renal or gastrointestinal (GI) losses [2]. Those with a high predisposition for the malabsorption of magnesium include, but are not limited to, a history of gastric sleeve, inflammatory bowel disorders, diabetes, alcohol use disorder, inherited tubular disorders (Bartter syndrome and Gitelman’s syndrome), and familial hypomagnesemia with hypercalciuria and nephrocalcinosis [1,2]. Additionally, some medications interfere with magnesium absorption, while others enhance magnesium excretion by the kidneys. This case highlights a commonly used group of medications, proton pump inhibitors (PPIs), with an uncommonly observed side effect of profound symptomatic hypomagnesemia.

## Case presentation

We present a case of a 68-year-old male with a past medical history of hypertension, hyperlipidemia, diabetes mellitus type 2, gastroesophageal reflux disease (GERD), prior alcohol abuse, and benign tremor who presented to the emergency department after a syncopal episode and profound weakness, leading to difficulties in moving.

The patient was admitted for a syncopal workup. He reported an unwitnessed loss of consciousness with an unknown downtime. Upon waking up, he felt lightheaded and was unable to move his lower extremities, arms, and torso. He denied any prodromal symptoms of flushing, the feeling of warmth, or palpitations preceding the syncopal episode. A review of systems was further negative for fever, chills, night sweats, weight loss, change in bowel habits, facial paralysis, slurring of speech, visual changes, or any focal neurological deficit. No pertinent alcohol use was noted leading up to the presenting events.

The patient reported home medications of atorvastatin daily, omeprazole (40 mg twice daily, for at least three years), metformin, glimepiride, and propranolol. He did not report any recent diuretic use. He denied any pertinent surgical history. Family history was significant for heart disease in the mother and father. His social history was remarkable for prior alcohol abuse, but he denies tobacco or recreational drug abuse. On admission, he was hemodynamically stable. Significant laboratory findings are in Table 1 and include an elevated white blood cell count, glucose, and parathyroid hormone and multiple electrolyte derangements including decreased potassium, calcium, and magnesium.

**Table 1:** Abnormal laboratory findings showing profoundly low levels of magnesium with concurrent effects of low calcium and potassium.

	Laboratory Value	Reference Range
White blood cell (WBC) count	10.5k/mm^3^	3.7-10.1k/mm^3^
Glucose	220 mg/dL	74-106 mg/dL
Potassium	3.2 mmol/L	3.5-5.1 mmol/L
Calcium	6.3 mg/dL	8.4-10.2 mg/dL
Magnesium	<0.20 mg/dL	1.6-2.3 mg/dL
Parathyroid hormone (intact)	189.5 pg/mL	18.5-88.0 pg/mL

Computed tomography (CT) of the abdomen, pelvis, and head was without acute abnormalities. Additionally, carotid Doppler and magnetic resonance imaging (MRI) of the brain were unremarkable for acute changes. Echocardiography was performed and revealed an ejection fraction of 55%-60%, mild mitral regurgitation, and no wall motion abnormalities. Electrocardiography showed sinus bradycardia and marked sinus arrhythmia. Given the concerns for the arrhythmogenic cause of syncope secondary to profound electrolyte derangements, he was placed on telemetry and given intravenous normal saline, magnesium sulfate, potassium chloride, and calcium gluconate. Additionally, his home omeprazole was held.

The next morning, he was still exhibiting mild lightheadedness upon standing. The patient had a negative orthostatic pulse and blood pressure changes. Otherwise, he reported that his lower extremity weakness had markedly improved. He was given a total of 6 g of intravenous magnesium sulfate on admission and started on 400 mg twice daily of magnesium oxide thereafter. The workup was negative for GI losses of magnesium as the patient did not report ongoing diarrhea. He also did not have any symptoms of malabsorption. The only plausible culprit for the low magnesium appeared to be omeprazole owing to the fact that the patient’s age rules out new-onset genetic causes of hypomagnesemia. He continued to improve over his hospital course and walked out of the hospital four days after admission, with recommendations to discontinue omeprazole and use alternative medications for acid reflux such as histamine blockers. He was referred to gastroenterology specialists for a follow-up upper endoscopy and advised to utilize a wedge pillow at night to help with reflux symptoms.

## Discussion

Magnesium is an essential divalent ion responsible for multiple cellular functions such as cell membrane depolarization, protein synthesis, and enzymatic reactions. Thus, when intracellular levels of magnesium are depleted, cellular functions are interrupted. Low serum levels of magnesium of less than 1.46 mg/dL lead to clinically significant signs and symptoms [1,2]. The clinical symptoms include neuromuscular hyperexcitability leading to seizures, tremors, or fasciculations. However, life-threatening consequences of hypomagnesemia arise in the electrical conduction of the heart, resulting in atrial and ventricular arrhythmias, such as torsades de pointes [3,4]. The mechanism of hypomagnesemia leading to electrical hyperexcitability is multifactorial and involves other electrolytes. Magnesium regulates the metabolism of potassium and calcium; thus, low levels of magnesium should trigger an investigation into these electrolytes. Given the fact that magnesium is not routinely measured during basic metabolic panels, this case highlights the importance of checking this electrolyte any time symptoms or signs suggest cell membrane electrical activity might be abnormal.

Hypomagnesemia is a rare cause of syncope, and our case highlights one such clinical scenario where the magnesium was reported at <0.20 mg/dL. In these scenarios, clinicians are forced to ask about the analysis used by laboratories to determine the cutoff at which serum electrolytes such as magnesium are no longer detectable. The analysis of magnesium can be measured in multiple ways including the serum (preferred method), heparinized plasma, or urine by photometric assays that use colorimetric reactions. The measurement of serum magnesium by an ion-selective electrode more accurately assesses the total body magnesium. In our facility, the Vitros XT 7600 (Ortho Clinical Diagnostics, Raritan, NJ) is utilized, which is an integrated analyzer system that uses an ion-selective electrode that meets the gold standard of testing. The linearity range for magnesium at our facility is 0.20-10.0 mg/dL, which is standard across laboratories in the United States [5]. However, different analyzers do exist, and the differences in linearity are variable. Thus, it is important to know how your laboratory detects and analyzes low serum electrolytes, especially when there are clinical implications such as symptoms consistent with depleted levels of the electrolyte in question. In our case, the patient had bradycardia and prolonged QTc, which can precipitate torsades de pointes.

When a patient presents with low serum magnesium, there are multiple environmental factors centered on conditions that lead to poor dietary intake or poor absorption of magnesium. Alcohol use disorder causes a multitude of electrolyte imbalances but specifically results in hypomagnesemia. Poor dietary intake as seen in patients receiving total parenteral nutrition or undergoing cancer treatments is associated with low serum magnesium levels [1,6]. Magnesium can be associated with gastrointestinal losses related to diarrhea, pancreatitis, gastric bypass, or hungry bone syndrome [6]. An often overlooked cause of hypomagnesemia is that medications, proton pump inhibitors, have been shown to cause low magnesium levels due to the impaired absorption of magnesium [3]. Other medications include loop and thiazide diuretics, aminoglycoside antibiotics, and laxative abuse [1].

Although secondary causes of hypomagnesemia are more common, genetic conditions can contribute to low magnesium. The genetic causes are divided into four main categories. These categories are hypercalciuric hypomagnesemia, Gitelman-like hypomagnesemia, mitochondrial hypomagnesemia, and others [7]. Gitelman-like hypomagnesemia is associated with the atrophy of the descending collecting tubule within the kidneys. This impairs the tubule’s ability to reabsorb magnesium, thus leading to low serum levels [7]. Mitochondrial hypomagnesemia, as the name implies, is an issue within the mitochondria itself. Unfortunately, the mechanism for how this category presents with low magnesium levels is still relatively unknown. Mitochondrial diseases with genetic mutations seen in Kearns-Sayre syndrome, seryl-tRNA synthetase 2 (SARS2), and mitochondrially encoded tRNA isoleucine (MT-TI) are associated with hypomagnesemia. The category of others is notable for genetic mutations that impair the epithelial magnesium channel within the colon and descending collecting tubule.

**Figure 1:**
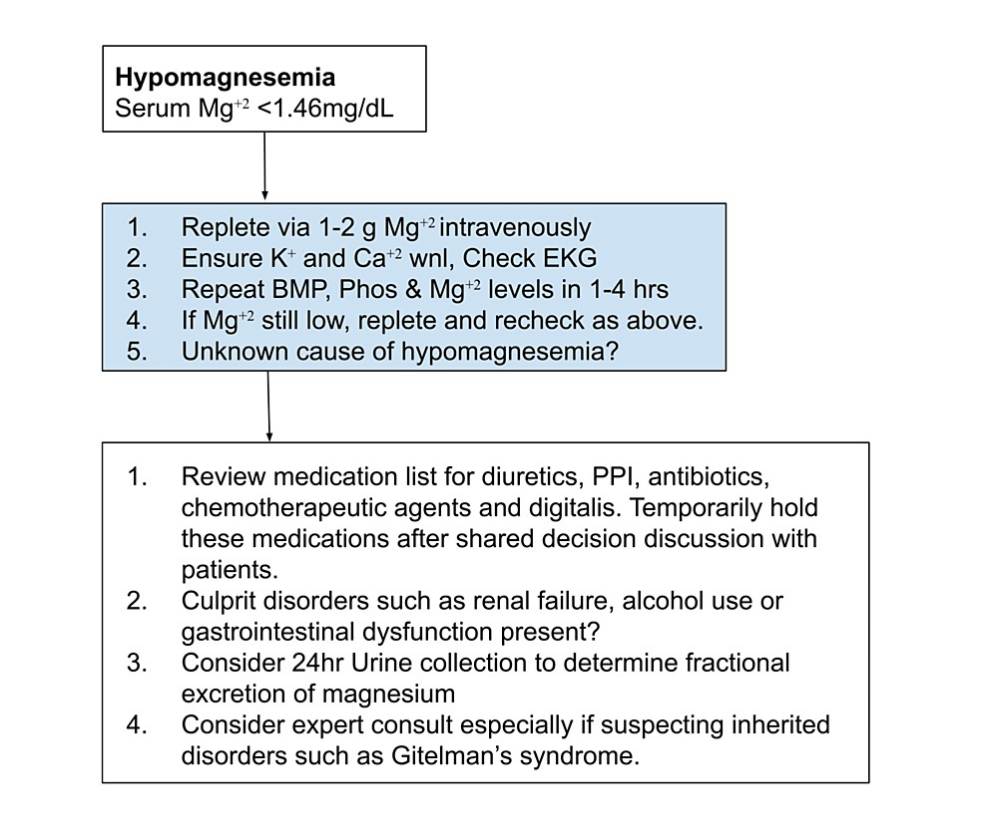
Proposed management algorithm for hypomagnesemia, a critical electrolyte abnormality that can lead to severe clinical consequences if not addressed. wnl, within normal limit; PPI, proton pump inhibitor; BMP, basic metabolic panel

In patients with clinically significant hypomagnesemia, it is important to replete the ion carefully, taking into consideration the patient’s renal function [8-10]. Based on a review of the current literature, a proposed algorithm for magnesium repletion is shown in Figure 1. In an emergent situation, 1-2 g of magnesium can be given over a 15-minute period [1,8]. In non-emergent situations, the patient can be given 4-8 g of magnesium over the course of 12-24 hours [1]. In a patient with decreased renal function, it is imperative that we reduce the dose of magnesium, as patients are at an increased risk for hypermagnesemia [1]. Finally, it is important that we recheck magnesium levels to ensure adequate treatment and continue treating for two days after the level normalizes [1]. Of note, patients who are able to tolerate oral intake and with chronically low magnesium levels can also supplement their magnesium intake via foods, such as nuts, halibut, and spinach [1].

## Conclusions

Magnesium is often called the forgotten cation; however, symptoms of hypomagnesemia can be life-threatening. Hypomagnesemia can result in disturbances in every organ system and even cause fatal arrhythmias and sudden death. Diagnosis is achieved with a high degree of clinical suspicion, thorough history taking, medication reconciliation, and measurement of magnesium with an ion-selective electrode. The ion-selective electrode provides the highest diagnostic yield in accurately diagnosing dangerously and clinically significant low levels of serum magnesium. It is important to recognize that some medications are more likely to increase the risk of magnesium malabsorption such as proton pump inhibitors. If a culprit medication is discovered, it should be discontinued immediately. Management should be geared toward immediate repletion and repeating the serum levels to ensure that serum magnesium levels improve or show an appropriate response. As such, the recognition of this deficiency is crucial in the clinical setting in order to implement immediate repletion therapy in hopes of circumventing the devastating life-threatening consequences that can result.
